# Emerging roles of circRNAs in leukemia and the clinical prospects: An update

**DOI:** 10.1002/iid3.725

**Published:** 2022-12-29

**Authors:** Wei Deng, Rong Chao, Shengdong Zhu

**Affiliations:** ^1^ Department of Pediatric General Internal Medicine Gansu Provincial Maternity and Child‐Care Hospital Lanzhou City Gansu Province People's Republic of China

**Keywords:** acute lymphoblastic leukemia, circRNAs, diagnosis, drug resistance, leukemia

## Abstract

**Background:**

Circular RNAs (circRNAs) are a new category of endogenous non‐protein coding RNAs (ncRNAs), and show the characteristics of high conservation, stability, and tissue specificity. Due to rapid advances in next‐generation sequencing and transcriptome profiling technologies, circRNAs have been widely discovered in many organisms and participated in the development and progress of a variety of diseases. As a type of molecular sponge, circRNAs mainly absorb micro RNAs competitively and interplay with RNA‐binding proteins to modulate the splicing as well as transcription of target genes.

**Methods:**

This review is based on a literature search using the Medline database. Search terms used were “circular RNAs and leukemia,” “circRNAs and leukemia,” “circRNAs and acute lymphoblastic leukemia,” “circRNAs and chronic lymphoblastic leukemia,” “circRNAs and acute myeloid leukemia,” “circRNAs and chronic myeloid leukemia,” and “circRNAs, biomarker, and hematological system.”

**Results:**

CircRNAs have been proven as potential biomarkers and therapeutic targets in a variety of tumors. Recent research has found that circRNAs aberrantly exist in hematological cancers, especially leukemia, and are significantly associated with the incidence, progress, and metastasis of diseases as well as the prognosis of patients.

**Conclusion:**

The current work summarizes the latest findings on circRNAs in various types of leukemia, aiming to propose prospective therapies and new drug screening methods for the treatment of leukemia.

## Graphical abstract

The current work reviews the latest research on circular RNAs in various types of leukemia, aiming to provide promising therapeutic methods and novel drug screening strategies for leukemia.

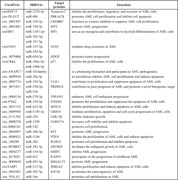

## 1 INTRODUCTION

Leukemia is a kind of malignancy with clonal hematopoietic stem cell disorders. Depending on the extent of differentiation and maturation of leukemia as well as its progressive course, leukemia can be categorized into acute or chronic leukemia.^1^ Acute leukemia is classified into acute myeloid leukemia (AML) and acute lymphoblastic leukemia (ALL) according to the main cells involved. Chronic leukemia is classified as chronic myeloid leukemia (CML) and chronic lymphoblastic leukemia (CLL). Over the past years, the life quality of leukemia patients has been greatly improved with the strengthening of supportive therapies, the combined use of multiple chemotherapeutic drugs, the high‐dose chemotherapeutic regiments, and the clinical application of hematopoietic stem cell transplantation.^2^, ^3^ However, there are certain leukemia cases, for instance, acute promyelocytic leukemia (APL) patients with mutations in PML‐RARα, NPM1, and FLT3‐ITD fusion genes, and CML patients with BCR‐ABL1 gene, show significant resistance to chemotherapeutic drugs, leading to disease progression and even death.^4^, ^5^ The research on the clinical diagnosis, treatment options, prognosis evaluation, and monitoring of leukemia has entered the stage of molecular biology. The mechanism underlying the proliferation, differentiation, and apoptosis of leukemia cells is being gradually understood. Certain nonprotein coding RNAs (ncRNAs) have been confirmed as key molecules participating in the pathogenesis of leukemia, their abnormal expression might be used to classify leukemia subtypes, evaluate the prognosis, as well as predict the response to chemotherapy.^6^ Circular RNAs (circRNAs) are a group of recently discovered ncRNAs associated with many diseases, especially tumors. CircRNAs are found to regulate the pathogenesis of leukemia mainly as a molecular sponge of specific micro RNAs (miRNAs), and the role and regulation of circRNAs in the proliferation, differentiation, metastasis, and chemotherapy resistance of leukemia cells are gradually being recognized.^7^

## 2 METHODS

This review is based on a literature search using the Medline database. Search terms used were “circular RNAs and leukemia,” “circRNAs and leukemia,” “circRNAs and acute lymphoblastic leukemia,” “circRNAs and chronic lymphoblastic leukemia,” “circRNAs and acute myeloid leukemia,” “circRNAs and chronic myeloid leukemia,” and “circRNAs, biomarker, and hematological system.”

## 3 THE CHARACTERISTICS AND REGULATION OF CIRCRNAS

As a novel noncoding gene fragment, circRNAs participate in various biosynthetic courses as well as the occurrence of diseases. Compared with other linear RNA fragments, circRNAs do not have the 5′‐terminal cap structure or the 3′‐end polyA‐tail structure, instead, circRNAs are closed‐loop structures formed by covalent bonds.^8^ CircRNAs extensively exist in a variety of tissues and organs, they indirectly modulate gene expression via regulating gene transcription, protein translation, and miRNA functions.^9^ CircRNAs were first discovered in viroids and have long been considered as the by‐products resulting from RNA aberrant splicing without specific biological functions.^10^ Now, circRNAs are shown to take part in numerous biophysiological courses such as growth, development, and aging in eukaryotes.^9^

CircRNAs are primarily derived from protein‐coding exons, they are noncoding sequences formed via reverse shearing of pre‐mRNAs during transcription.^11^ The process of circRNA synthesis usually involves the transcription and shearing of parent genes and competes with the linear shearing mechanism.^11^ There are two main ways to obtain circRNAs, one is through the exon cyclization of the protein‐coding genes, and the other is through the reverse splicing of intron‐driven cyclization.^12^ The source sequences of circRNAs include intron region, untranslated region, intergenic region, and reverse sequence known to be transcribed.^12^

CircRNAs are abnormally expressed in a variety of diseases. CircRNAs act as a “sponge” to specifically adsorb miRNAs, prevent them from binding to the target genes and thus inhibit target gene regulation.^13^ The miRNA binding sites on circRNAs can bind miRNAs and negatively modulate them, regulate the target genes of specific miRNAs, and further regulate mRNA transcription and protein translation.^13^ For example, the expression level of circRNA‐5692 was downregulated in hepatocellular carcinoma (HCC) tissues, while its overexpression was found to attenuate the malignancy in HCC cells.^14^ Bioinformatic analysis predicted that circRNA‐5692 could interplay with miR‐328‐5p, which targeted DAB2IP mRNA, forming a circRNA‐miRNA‐mRNA complex. In vivo study showed that the overexpression of circRNA‐5692 suppressed xenograft HCC tumor growth via inhibiting miR‐328‐5p to elevate DAB2IP level, indicating suppressive effects of circRNA‐5692/miR‐328‐5p/DAB2IP network on HCC development.^14^ Another study found that circ‐MAPK4 was downregulated during the early neuronal differentiation. However, circ‐MAPK4 in gliomas was found to increase and coincide with the pathological grade and stage of gliomas by promoting glioma cell survival and inhibiting apoptosis. MiR‐125a‐3p, a tumor‐suppressive miRNA by targeting p38/MAPK, was found to increase after suppressing circ‐MAPK4, and it could be pulled down by circ‐MAPK4. Inhibition of miR‐125a‐3p was found to partially rescue p38/MAPK phosphorylation and apoptosis by circ‐MAPK4 knockdown, suggesting circ‐MAPK4 as an important modulator in the survival and apoptosis of glioma cells by regulating miR‐125a‐3p/p38/MAPK pathway.^15^

## 4 CIRCRNAS IN THE HEMATOLOGICAL SYSTEM

CircRNAs are widely expressed in the whole blood, platelets, and exosomes in the hematological system.^16^ CircRNAs are closely associated with the growth, aging, and disease process of the body, exerting key roles in the regulation of the blood system. Plenty of circRNAs were found to exist in the whole blood or blood components, which might function as key targets to affect disease processes and potential biomarkers for accurate diagnosis.^17^ A recent study by whole transcriptome sequencing found that 450 circRNAs were differentially expressed in peripheral blood between schizophrenia patients and healthy people.^18^ Also, the expression patterns and related pathways of circRNA, circRNA‐miRNA‐mRNA competitive endogenous RNAs (ceRNA), and RNA‐binding protein network demonstrate the possible function of circRNAs in schizophrenia pathogenesis.^18^ Therefore, abnormal expression of circRNAs in the whole blood might predict the occurrence and progression of certain diseases. Platelets are small, irregularly shaped cell fragments derived from mature megakaryocytes that take a crucial part in hemostasis, coagulation, and angiogenesis. A recent work detected abundant circRNAs in platelet concentrates by RNA‐sequencing.^19^ Among them, the expression of 198 circRNAs changed dramatically with 13 circRNAs changing continuously during platelet storage.^19^ The enrichment of circRNAs varies among different blood components. For example, 17−188 times more circRNAs were present in human platelets relative to nucleated tissues, while exons within circRNAs were concentrated ~12.7 times in platelets compared with nucleated cells.^20^ Moreover, the diversity of circRNAs varies among different blood cell types. A comparison of circRNAome from B‐cells, T‐cells, and monocytes in healthy subjects by rRNA‐depleted total RNA‐seq revealed cell‐specific circRNA expression patterns and alternative circularization.^21^ However, only a few studies focusing circRNAs in hematological malignancy have been conducted, it is necessary to further reveal the interaction between circRNAs in the platelets with miRNAs and mRNAs to provide a new therapeutic strategy. At present, exosomes have been found as novel media for information transmission between cells and attracted more and more attention. Exosomes in the plasma are disc‐shaped bilayer lipid vesicles containing nucleic acids and proteins, they are generated in various hemocytes and tumor cells.^22^ Exosomes derived from platelets have been found rich in circRNAs, which are transported to recipient cells via exosomes and further regulate the biological activities in recipient cells.^22^, ^23^

## 5 CIRCRNAS AND LEUKEMIA

### 5.1 CircRNAs in AML

AML belongs to a hematological malignancy featured with abnormal proliferation of immature myeloid cells, which affect the normal formation of bone‐marrow hematopoietic cells.^24^ AML is characterized by high heterogeneity, rapid progression, a high rate of relapse, and a low rate of long‐term survival.^24^ AML's incidence increases progressively with age, it is the most frequent acute myeloid primitive cell malignancy in adults. At present, it is believed that the pathogenesis of AML and the resistance to chemotherapeutic drugs are the result of multiple gene mutations via various mechanisms. AML's prognosis also varies due to different risk stratification of the disease.^25^ A lot of cytogenetic abnormalities contributing to AML are revealed in succession, including t(8; 21), t(15; 17), and t(9; 11) chromosomal translocations, which produce fusion proteins RUNX1‐RUNX1T1, PML‐RARα, and MLL‐AF49, respectively.^26^ Fusion circRNAs (f‐circRNAs) have been confirmed to originate from fusion genes generated via chromosomal translocations. Research found that cancer‐associated chromosomal translocations could cause the production of f‐circRNAs, which lead to cell transformation, increase cell activity, and enhance therapeutic resistance, showing tumor‐promoting effects.^27^

CircRNAs exert key roles in AML. The dysregulation of circRNA expression may result from the abnormal damage in leukemia cells and participate in AML pathogenesis and the mechanism of chemotherapeutic resistance. circMYBL2 was found to exist more abundantly in AML patients carrying FLT3‐ITD mutations.^28^ CircMYBL2 knockdown specifically suppressed FLT3‐ITD AML proliferation and promoted its differentiation, and also damaged cytoactivity of inhibitor‐resistant FLT3‐ITD‐positive AML,^7^ suggesting circMYBL2 as a possible target for FLT3‐ITD AML treatment.^28^ circRNF220 was found to abundantly accumulate in peripheral blood and bone marrow of pediatric patients with AML.^29^ The abnormally high level of circRNF220 was suggested to be an adverse and independent prognostic marker for AML reoccurrence, its knockdown specifically inhibited AML proliferation and facilitated its apoptosis via interacting with miR‐30a to increase MYSM1 and IER2 expression.^29^ circBCL11B, a newly identified circRNA, was found to exclusively present in AML patients.^30^ CircBCL11B knockdown inhibited AML proliferation and promoted cell death, indicating that circBCL11B might be a new functionally relevant modulator during AML pathogenesis.^30^ Moreover, circ_0002483 knockdown was found to repress proliferation and promote cycle arrest and apoptosis by regulating miR‐758‐3p/MYC in AML cells.^31^ Recent findings on the regulation of circRNA/miRNA/target protein network in AML have been shown in Table 1.

**Table 1:** The regulation of circRNA/miRNA/target protein network in acute myeloid leukemia (AML)

CircRNAs	MiRNAs	Target proteins	Functions
circRNF13	miR‐1224‐5p	Tenascin‐C	Inhibits the proliferation, migration, and invasion of AML cells
circ‐DLEU2	miR‐496	PRKACB	Promotes AML cell proliferation and inhibits cell apoptosis
circ_0001947	miR‐329‐5p	CREBRF	Functions as a tumor inhibitor to suppress AML cell proliferation
circ_0002483	miR‐758‐3p	MYC	Promote AML progression
circSPI1	miR‐1307‐3p/	SPI1	Acts as an oncogene and contributes to myeloid differentiation of AML cells
circPAN3	miR‐382‐5p/	XIAP	Mediates drug resistance in AML
circ_0079480	miR‐767‐5p	HDGF	Promotes tumor progression
circCRKL	miR‐153‐5p/	p27	Inhibits the proliferation of AML cells
circ‐ANAPC7	miR‐183‐5p	/	Is a promising biomarker and participates in AML pathogenesis
circ_0009910	miR‐654‐3p	/	Its knockdown inhibits AML cell proliferation and induces apoptosis
circ_0005774	miR‐196a‐5p/	ULK1	Contributes to proliferation and suppresses apoptosis of AML cells
circ_0075451	miR‐196b‐5p	PRDM16	Contributes to poor prognosis of AML and presents a novel therapeutic target
circ_0004136	miR‐181family	TSPAN3	Enhances AML cell malignant progression
circ‐PTK2	miR‐20a‐5p	FOXM1	Promotes the proliferation and suppresses the apoptosis of AML cells
circ_0012152	miR‐192‐5p	SOX12	Inhibits proliferation and induces apoptosis in AML cells
circ_0009910	miR‐330‐5p/	GRB10	Mediates proliferation, apoptosis, and cell cycle progression of AML cells
circ_0121582	miR‐326	GSK‐3β	Inhibits leukemia growth
circ_0000370	miR‐570‐3p	S100A7A	Increases cell viability and inhibits apoptosis
circ‐0004136	miR‐330‐5p	/	Promotes cell proliferation
circ_0044907	miR‐625‐5p	KIT	Promotes AML progression
circ_0040823	miR‐5195‐3p	PTEN	Inhibits the proliferation of AML cells and induces apoptosis
circ_100290	miR‐224	RAB10	Promotes cell proliferation and inhibits apoptosis
circ‐SFMBT2	miR‐1299	ZBTB20	Facilitates the malignant growth of AML cells
circ_0004277	miR‐142	SSBP2	Inhibits AML progression
circ_KCNQ5	miR‐186‐5p	RAB10	Participates in the progression of childhood AML
circ_0009910	miR‐516b	B4GALT5	Promote AML progression
circTASP1	miR‐203	HMGA2	Inhibits proliferation and induces apoptosis of AML cells
circ_0003602	miR‐582‐3p	IGF1R	Accelerates the tumorigenicity of AML
circ_POLA2	miR‐134‐5p	/	Promotes cell proliferation in AML
	miR‐622		
	miR‐491‐5p		
	miR‐515‐5p		
	miR‐502‐5p		
	miR‐34a		

Abbreviations: B4GALT5, beta‐1,4 galactosyltransferase V; circRNAs, circular RNAs; CREBRF, CREB3 regulatory factor; FOXM1, forkhead box M1; GRB10, growth factor receptor‐bound protein 10; GSK‐3β, glycogen synthase kinase‐3β; HDGF, hepatoma‐derived growth factor; HMGA2, high mobility group protein A2; IGF1R, insulin‐like growth factor 1 receptor; miRNAs, micro RNAs; PRDM16, PR domain‐containing protein 16; PRKACB, protein kinase CAMP‐activated catalytic subunit beta; PTEN, phosphatase and tensin homolog deleted on chromosome 10; S100A7A, S100 calcium‐binding protein A7A; SOX12, sex‐determining region Y‐box 12; SPI1, salmonella pathogenicity island 1; SSBP2, single‐stranded DNA binding protein 2; TSPAN3, tetraspanin‐3; ULK1, UNC‐51‐like kinase 1; XIAP, X‐linked inhibitor of apoptosis; ZBTB20, zinc finger and BTB domain containing 20.

APL is an uncommon type of AML featured with the formation of PML‐RARα fusion protein.^32^ APL is highly responsive to all‐trans‐retinoic acid (ATRA) therapy and promotes the differentiation and maturation of leukemia.^4^, ^33^ The expression of circ‐HIPK2 in APL patients was found to be dramatically less than that in normal peripheral blood mononuclear cells (PBMCs) or other AML subtypes, suggesting circ‐HIPK2 as a potential biomarker for APL.^34^ Mechanically, circ‐HIPK2 influenced ATRA‐induced differentiation in APL cells via sponging miR‐124‐3p.^34^

### 5.2 CircRNAs in CML

CML is a malignant myeloid proliferative tumor occurring in pluripotent hematopoietic stem cells with an annual incidence of 1−2 per 100,000 people.^35^ CML shows a significant increase of immature peripheral blood granulocytes and is featured by the typical genetic characteristics of t(9; 22) (q34; q11) mutual translocation.^35^ It gives rise to the generation of the *BCR‐ABL* fusion gene and expression of the BCR‐ABL1 fusion protein,^35^, ^36^ which is a tyrosine kinase mediating the phosphorylation and activation of crucial molecules involved in survival and growth of bone marrow progenitor cells. Imatinib belongs to tyrosine kinase inhibitor (TKI) and is a targeted drug for CML treatment, it can make some CML patients get significant remission.^37^ However, there are still 8%−13% of CML patients showing severe resistance to imatinib, which seriously decreases the drug efficacy. The results confirmed that the BCR‐ABL fusion gene and its abnormal expression may increase the resistance of CML cells to imatinib.^38^ Moreover, mutations in the BCR‐ABL1 kinase region may also enhance drug resistance. A current study discovered a new f‐circRNA, named F‐circBA1, in K562 and K562/G01 cells.^39^ F‐circBA1 knockdown by shRNA arrested cell cycle and proliferation and repressed leukemogenesis via interacting with miR‐148‐3p to decrease CDC25B expression.^39^ F‐circBA1 has also been detected in certain BCR‐ABL‐positive CML patients, indicating the existence of F‐circBA1 and its oncogenic function in CML.^39^

In addition to the BCR‐ABL fusion gene, there are also certain ncRNAs that regulate the transcription and translation of specific genes and participate in the progress of CML. The upregulation or downregulation of circRNAs takes an important part in assessing the prognosis, pathogenesis, and resistance to chemotherapeutic drugs of CML.^40^ Circ_0058493 was significantly overexpressed in PBMCs of CML patients with poor clinical response to imatinib.^41^ Knockdown of circ_0058493 markedly repressed the growth of imatinib‐resistant CML cells probably via acting as a molecular sponge to miR‐548b‐3p, suggesting that circ_0058493 in PBMCs might be a useful prognostic biomarker as well as a therapeutic target for CML.^41^ CircHIPK3 was also significantly elevated in PBMCs and the serum in CML patients with poor outcomes. Further loss‐of‐function studies demonstrated circHIPK3's oncogenic effect on CML.^42^ The circ_0080145 level was raised in imatinib‐resistant CML patients and cells. Circ_0080145 knockdown not only suppressed imatinib resistance, cell proliferation, glycolysis, and induced apoptosis in imatinib‐resistant CML in vitro but also restrained tumor growth and imatinib resistance in vivo via modulating miR‐326/PPFIA1 axis.^43^ However, the overall dynamic regulatory network of circRNAs related to CML disease progression and TKI resistance, as well as the involved mechanisms still need to be further elucidated. Recent findings on the regulation of circRNA/miRNA/target protein networks in CML have been shown in Table 2.

**Table 2:** The regulation of circRNA/miRNA/target protein network in chronic myeloid leukemia (CML)

CircRNAs	MiRNAs	Target proteins	Functions
circ_0080145	miR‐29b	/	Regulates CML cell proliferation
circ_0009910	miR‐34a‐5p	ULK1	Accelerates imatinib‐resistance in CML cells by modulating autophagy
circ_0080145	miR‐203	ABL1	Responsible for the development of imatinib chemoresistance in CML
circ 0051886	miR‐637	ABL1	Responsible for the development of imatinib chemoresistance in CML
circ_0058493	miR‐548b‐3p	/	As a novel biomarker for Imatinib‐resistant CML
F‐circBA1	miR‐148‐3p	CDC25B	Displays an oncogenic role in CML cells
circ_0080145	miR‐326	PPFIA1	Enhances imatinib resistance in CML

Abbreviations: ABL1, abelson 1; CDC25B, cell cycle division 25B; circRNAs, circular RNAs; miRNAs, micro RNAs; PPFIA1, liprin‐alpha1; ULK1, UNC‐51‐like kinase 1.

### 5.3 CircRNAs in ALL

ALL is a common malignant tumor in childhood, most of which are genetically heterogeneous tumors induced by malignant proliferation of lymphoid progenitor cells with gene mutations, accompanied by varying degrees of development or differentiation arrest.^44^ Currently drug therapy can achieve a high survival rate, however, 15%−20% of pediatric patients may develop relapse.^45^ Many studies indicate that circRNAs positively participated in ALL pathogenesis, showing promising diagnostic and therapeutic values. For example, circADD2 was a tumor suppressor and remarkably downregulated in ALL tissues and cell lines.^46^ CircADD2 overexpression was found to inhibit cell proliferation and promote apoptosis via directly binding to miR‐149‐5p to further downregulate AKT2, indicating the possible function of circADD2 as an indicator for ALL diagnosis and treatment.^46^ The upregulation of circ‐0000745 was found in Kasumi‐1 and KG‐1ALL cell lines, which significantly enhanced cell proliferation and reduced cellular apoptosis by activating the ERK pathway.^47^ Circ_0000745 was found to regulate NOTCH1‐mediated cell proliferation and apoptosis in pediatric T‐ALL via adsorbing miR‐193b‐3p,^48^ and promote ALL development by modulating miR‐494‐3p/NET1.^49^ CircPVT1 was found to participate in T‐ALL progression through miR‐30e/DLL4 axis,^50^ and facilitate the proliferation and invasion via miR‐125b modulation of NF‐κB in ALL,^51^ representing a promising target for T‐ALL therapy. In addition, the expression profile and tissue/cell/development stage‐specific expression pattern of circRNAs were potential indicators to designate AL into different subtypes. For instance, expression signatures of Hsa_circ_0012152 and Hsa_circ_0001857 could be used to accurately distinguish ALL from AML by microarray.^52^ A large‐scale circRNA deregulation was confirmed in T‐ALL by CirComPara analysis of RNA‐seq data.^53^ Recent findings on the regulation of circRNA/miRNA/target protein network in ALL have been shown in Table 3.

**Table 3:** The regulation of circRNA/miRNA/target protein network in acute lymphoblastic leukemia (ALL)

CircRNAs	MiRNAs	Target proteins	Functions
circ_0000094	miR‐223‐3p	FBW7 AKT2	Restrains T cell ALL progression
circADD2	miR‐149‐5p	ERK1/2	As a tumor suppressor and a potential biomarker for ALL diagnosis or treatment
circ‐0000745	/	DLL4	Enhances cell proliferation and viability
circPVT1	miR‐30e	NOTCH1	Participates in the progression of T‐ALL
circ_0000745	miR‐193b‐3p	SOX4 Reelin	Promotes cell proliferation and curbs cell apoptosis
circPRKCI	miR‐20a‐5p	NET1	Contributes to malignant progression of T‐ALL
circ‐PRKDC	miR‐653‐5p	NF‐κB	Its depletion enhances autophagy and apoptosis in T‐ALL
circ_0000745	miR‐494‐3p	GRα	Promotes ALL progression
circPVT1	miR‐125b		Promotes ALL cell proliferation and invading
circ_0000143	miR‐142‐3p		Its downregulation facilitates the progression of T‐ALL

Abbreviations: AKT2, serine/threonine kinase 2; circRNAs, circular RNAs; DLL4, delta‐like 4; ERK1/2, extracellular signal‐regulated kinase 1/2; FBW7, F‐box and WD repeat domain‐containing protein 7; GRα, glucocorticoid receptor alpha; miRNAs, micro RNAs; NET1, neuroepithelial cell transforming 1; NF‐κB, nuclear factor‐kappa B; SOX4, SRY‐related high‐mobility‐group box 4.

### 5.4 CircRNAs in CLL

CLL is a chronic lymphoproliferative disease of B cells, featured by the continuous accumulation of monoclonal CD5^+^ B lymphocytes in bone marrow, peripheral blood, spleen, and lymph nodes.^54^ CLL shows relatively lower proliferative and higher antiapoptotic capabilities and is the most frequent type of adult leukemia in western countries.^55^ The annual incidence of CLL rises with age, with the prevalence of CLL among people over 65 years old up to 12.8 per 100,000 people. Recently, the dysregulation of circRNAs has been shown to contribute to CLL pathogenesis. A current study investigated expression of 13,368 cricRNAs in 21 CLL patients and found that regardless of the clinical, prognostic, or genetic characteristics, CLL cells show a distinct cricRNA profile different from that in normal B lymphocytes.^56^ Circ_0132266 was significantly reduced in CLL patient‐derived PBMCs in comparison to that from normal subjects, serving as the endogenous sponge for miR‐337‐3p to further regulate the expression of target gene PML and thus promote CLL progression.^57^ Circ‐RPL15 was found in the plasma and identified as a new oncogenic driver to accelerate the pathological process of CLL.^58^ Circ‐CBFB was shown to motivate CLL growth and reduce apoptosis via modulating the miR‐607/FZD3/Wnt/β‐catenin axis.^59^ circZNF91 was shown to promote the malignant phenotype in CLL by targeting the miR‐1283/WEE1 axis, which might serve as potential therapeutic targets.^60^ Recent findings on the regulation of circRNA/miRNA/target protein network in CLL have been shown in Table 4.

**Table 4:** The regulation of circRNA/miRNA/target protein network in chronic lymphoblastic leukemia (CLL)

CircRNAs	MiRNAs	Target proteins	Functions
circ_0132266	miR‐337‐3p	PML	Its downregulation promotes CLL cell viability
circ‐CBFB	miR‐607	FZD3	Promotes proliferation and inhibits apoptosis of CLL cells
circZNF91	miR‐1283	WEE1	Promotes the malignant phenotype of CLL cells

Abbreviations: CircRNAs, circular RNAs; FZD3, frizzled class receptor 3; miRNAs, micro RNAs; PML, promyelocytic leukemia protein; WEE1, WEE1‐like protein kinase.

Current studies mainly focus on circRNAs originating from the nuclear genome, however, the biological and clinical properties of mitochondrial genome‐derived (mt) circRNAs remain sparsely characterized, particularly in CLL. Mc‐COX2, a kind of mt‐circRNA, was abundantly presented in CLL patient‐derived plasma exosomes and positively related to leukemogenesis and deteriorated condition in CLL patients.^61^ Mc‐COX2 knockdown was shown to damage mitochondrial function, inhibit cell proliferation, promote cell apoptosis, and enhance antitumor efficacy of drugs, indicating its involvement in CLL development and prognosis.^61^

## 6 CONCLUSION AND PROSPECTS

Currently, the research on circRNAs has become a hot spot. CircRNAs can function as miRNA targets, transcription regulators, protein adapters, or protein translation factors, exerting numerous regulatory roles by sponging specific miRNAs. CircRNAs show high abundance and evolutionary conservation across species, characterized by tissue/cell‐specific expression. More importantly, circRNAs could be extensively detected in peripheral blood, presenting the feasibility as noninvasive biomarker. Therefore, circRNAs play latently important roles in the early diagnosis, prognostic evaluation, pathogenesis, and chemotherapeutic resistance of leukemia, as well as the proliferation, differentiation, and progression of leukemia cells.

However, compared with other linear RNAs, the research on circRNAs is still very limited, many problems need to be solved before their application in preclinical and clinical studies. For example, the current approaches for quantifying circRNAs have shown certain limitations that restrain the development of clinically applicable quantitative assays. A recent study analyzed the expression profile of circRNAs in several B cell malignancies via high‐throughput RNA‐seq, and quantified multiple differentially expressed circRNAs by NanoString technology.^62^ This study presented an enzyme‐free digital counting method that might serve as the new gold standard for the quantitative detection of circRNAs. Besides, the uniform database and naming criterion of circRNAs are not established, the annotations on circRNA functions are not detailed, and circRNA‐related regulatory mechanisms are not clear. Moreover, the feasibility of circRNAs as a definite biomarker of leukemia still needs more experimental verification. In the future, with the rapid progression in data‐driven algorithms, single‐cell sequencing, and microarray technologies, it is required to enlarge sample sizes and types to find more representative circRNAs as biomarkers, so as to improve the prognostic evaluation system of leukemia. At present, the research of circRNAs in the pathogenesis of leukemia is still in the initial stage, most of which are based on the hypothesis of ceRNAs and lack of sufficient novelty. With the discovery and identification of more circRNAs, as well as the confirmation of more functional experiments and clinical trials, the emergence of circRNA‐related targeting therapy of leukemia will be significantly accelerated, realizing the successful conversion of basic medicine to clinical practice. Therefore, circRNAs show huge potential and are expected in the clinical practice of leukemia. For example, following a risk assessment of leukemia based on the clinical characteristics, bone marrow cell morphologies, cytogenetics, immunophenotypes, as well as molecular abnormalities, clinicians can provide the optimal therapeutic approach or long‐term follow‐up plan for leukemia patients.

## AUTHOR CONTRIBUTIONS

**Deng Wei**: conceived, prepared, and revised the manuscript. **Deng Wei, Chao Rong, and Zhu Shengdong**: made the data acquisition and interpretation. All authors approved the final manuscript for publication.

## CONFLICT OF INTEREST

The authors declare no conflict of interest.

## Data Availability

Data are all available upon reasonable request.
